# Surgery and protontherapy in Grade I and II skull base chondrosarcoma: A comparative retrospective study

**DOI:** 10.1371/journal.pone.0208786

**Published:** 2018-12-17

**Authors:** François Simon, Loïc Feuvret, Damien Bresson, Jean-Pierre Guichard, Sophie El Zein, Anne-Laure Bernat, Moujahed Labidi, Valentin Calugaru, Sébastien Froelich, Philippe Herman, Benjamin Verillaud

**Affiliations:** 1 AP-HP, Hôpital Lariboisière, Department of Otorhinolaryngology and Paris Diderot University, Paris, France; 2 AP-HP, Hôpital Pitié-Salpêtrière, Department of Radiation Oncology and Pierre et Marie Curie University, Paris, France; 3 Institut Curie-Centre de protonthérapie d’Orsay, Department of Radiation Oncology and INSERM U61, Centre Universitaire, Orsay, France; 4 AP-HP, Hôpital Lariboisière, Department of Neurosurgery and Paris Diderot University, Paris, France; 5 AP-HP, Hôpital Lariboisière, Department of Radiology and Paris Diderot University, Paris, France; 6 AP-HP, Hôpital Lariboisière, Department of Pathology and Paris Diderot University, Paris, France; Roswell Park Cancer Institute, UNITED STATES

## Abstract

**Objective:**

Skull base chondrosarcoma is a rare tumour usually treated by surgery and proton therapy. However, as mortality rate is very low and treatment complications are frequent, a less aggressive therapeutic strategy could be considered. The objective of this study was to compare the results of surgery only vs surgery and adjuvant proton therapy, in terms of survival and treatment adverse effects, based on a retrospective series.

**Methods:**

Monocentric retrospective study at a tertiary care centre. All patients treated for a skull base grade I and II chondrosarcoma were included. We collected data concerning surgical and proton therapy treatment and up-to-date follow-up, including Common Terminology Criteria for Adverse Events (CTCAE) scores.

**Results:**

47 patients (23M/24F) were operated on between 2002 and 2015; mean age at diagnosis was 47 years-old (10–85). Petroclival and anterior skull base locations were found in 34 and 13 patients, respectively. Gross total resection was achieved in 17 cases (36%) and partial in 30 (64%). Adjuvant proton therapy (mean total dose 70 GyRBE,1.8 GyRBE/day) was administered in 23 cases. Overall mean follow-up was 91 months (7–182). Of the patients treated by surgery only, 8 (34%) experienced residual tumour progression (mean delay 51 months) and 5 received second-line proton therapy. Adjuvant proton therapy was associated with a significantly lower rate of relapse (11%; *p* = 0.01). There was no significant difference in 10-year disease specific survival between patients initially treated with or without adjuvant proton therapy (100% vs 89.8%, *p* = 0.14). Difference in high-grade toxicity was not statistically significant between patients in both groups (25% (7) vs 11% (5), *p* = 0.10). The most frequent adverse effect of proton therapy was sensorineural hearing loss (39%).

**Conclusion:**

Long-term disease specific survival was not significantly lower in patients without adjuvant proton therapy, but they experienced less adverse effects. We believe a surgery only strategy could be discussed, delaying as much as possible proton therapy in cases of relapse. Further prospective studies are needed to validate this more conservative strategy in skull base chondrosarcoma.

## Introduction

Chondrosarcoma (CSA) is a rare cartilaginous malignant tumour which, in the head and neck, can involve the sinonasal tract, the skull base (anterior skull base or petroclival suture), jaws or larynx. The tumour is classified by the World Health Organization in three grades, where grade III can often lead to metastasis and aggressive local recurrence [1]. The most frequent is grade II which, like Grade I, grows slowly and does not metastasise. [2–5]. Due to the rarity of the tumour, no randomized control trial has been conducted to compare treatment strategies. Also, most retrospective studies had to reach many years back (up to 30 or 40 years in some cases) to include a sufficient number of patients probably undermining surgical results [3, 6–9].

Unlike other CSA in the body, head and neck CSA cannot benefit from “en bloc” resections due to important surrounding structures, hence resection is often partial [3, 6, 10]. In skull base CSA, most authors agree to say that the best treatment to limit the risk of relapse is gross total surgical resection (GTR) followed by adjuvant proton therapy (PT) [6, 8, 10–12]. Many studies have shown that PT is an efficient method to improve the dose gradient between the gross tumour and surrounding structures [12–14]. However, PT still has adverse effects, due to possible damage to the brain stem, temporal lobe, optic chiasm, pituitary gland or inner ear [12]. In recent skull base CSA studies combining surgery and PT, the 10-year survival rate is excellent, over 85% [10, 12]. The current interrogation is whether it is possible to maintain this high survival rate while decreasing the side effects of the treatment.

The main objective of this retrospective study was to compare the outcome in terms of survival and toxicity of patients treated by surgery only and patients treated by surgery and adjuvant proton therapy.

## Materials and methods

This was a retrospective study of consecutive patients who underwent surgery in a tertiary referral centre from 2002 to 2015, corresponding to a period when endoscopic surgery and PT was routinely available and mastered by the medical team. The inclusion criteria were as follows: skull base CSA, surgical resection in our department and immunohistochemical confirmation of the diagnosis. Markers used were anti-brachyurea, anti-D240 and anti-PS100 antibodies (if not initially available, frozen samples were retrieved for immunohistology). Grade I and II CSA were included, as the treatment and prognosis are identical. All patients were operated on by a team of ENT, neurosurgeons or both, trained in skull base surgery, either by endoscopic surgery or open surgery. All patients were then eligible to PT. The general attitude was to treat residual CSA with PT, however after multidisciplinary discussion on a case-by-case basis, the strategy could vary, prompting this retrospective study. No other adjuvant treatment was discussed (including exclusive photon radiotherapy and chemotherapy).

When patients underwent PT, a standardised protocol was followed: 1.8 GyRBE (relative biological effectiveness) daily, five days a week for eight weeks, to deliver a total dose of 70 GyRBE [12]. The beams and apparatus characteristics have previously been described [12, 15]. If the full proton slot was not available, patients receive a combined proton/photon treatment (which happened to four patients in our study). The Proton treatment plans were optimised for the prescribed dose to reach ≥ 95% of the target volume. There were dose constraints on the optic nerve, anterior part of brainstem, spinal cord and contralateral ear (maximum dose 56, 64, 55 and 55 GyRBE respectively). The ipsilateral ear did not have dose constraints. The gross tumour volume (GTV) was delineated on post-operative MRI or CT-scans. The clinical target volume (CTV) included the GTV and 5- to 20-mm 3D margins that were manually corrected depending on suspected microscopic spread, natural barriers and anatomy. The planning target volumes were calculated by adding a 2- to 3-mm safety margins around the CTVs.

The patients’ medical records were reviewed for clinical, imagery, treatment and follow-up data. The latest clinical examination, MRI imagery and audiometric tests were studied. All patients were contacted by phone or by e-mail using standardised questions to collect missing data and up-to-date symptoms. Side-effects were graded according to the Common Terminology Criteria for Adverse Events, version 4.0 (CTCAE) [16]. The original data is available online (S1 Dataset).

This is a retrospective study and has been approved by our Institution Review Board (IRB), the CEORL (Evaluation Commission of Observational Research of the French Society of Otorhinolaryngology) and all clinical investigation have been conducted according to the principles expressed in the Declaration of Helsinki. Informed consent, written and oral, has been obtained from the participants.

Statistical analysis was made using XLstats (Addinsoft, New York, NY). Continuous data was summarised with means and range and compared using the nonparametric Mann-Whitney test. Categorical data was summarised with frequency counts and proportions and compared using the chi-square test. Disease Specific Survival (DSS) and progress-free survival (PFS) was determined using the Kaplan-Meier survival curves and were compared using the log-rank test. Deaths which were not due to CSA evolution or treatment were censored. The survival intervals were calculated from the date of the first surgery to the last follow-up. Binary and ordinal logistic regression tests were used to search for prognosis factors. A *p* value <.05 was considered significant.

## Results

Between 2002 and 2015, 47 consecutive patients were operated on in our institution for skull base CSA. Immunohistochemical pathology systematically confirmed the diagnosis. All were Grade II tumours except one Grade I (one patient with Grade III CSA had been managed over this period and had been excluded), there were no mesenchymal tumours. Two patients (4%) had received conventional photon radiotherapy and another 2 had been operated on prior to treatment in our institution. Patient characteristics are reported in Table 1. Overall, 34 patients (72%) had petroclival locations and mainly presented with intermittent and then permanent diplopia due to VI^th^ cranial nerve palsy; 13 patients (28%) had anterior locations and mainly presented with nasal obstruction.

**Table 1:** Patient characteristics (type of treatment and total population).

Characteristics	Surgery & Proton	Surgery only	*p*^a^	All patients
Number of patients	23 (49)	24 (51)	-	47 (100)
Sex				
Male	13 (57)	10 (41)	.*30*	23 (49)
Female	10 (43)	14 (59)		24 (51)
Age (years)	42 (12–69)	52 (10–85)	.*09*	47 (10–85)
Anatomical Localisation				
Anterior skull base	1 (4)	12 (50)	.*02*	13 (28)
Petroclival	22 (96)	12 (50)		34 (72)
Symptoms				
Diplopia	13 (57)	7 (29)	.*06*	20 (43)
Headache	8 (35)	4 (17)	.*15*	12 (26)
Nasal Obstruction	1 (4)	7 (29)	.*02*	8 (17)
ICA abutment	17 (74)	10 (42)	.*03*	27 (57)
Tumour Size (mm)	33 (19–67)	39 (15–70)	.*35*	36 (15–70)
Surgical approach				
Pterional	5 (22)	5 (21)	.*94*	10 (21)
Transcochlear	0 (0)	2 (8)	.*16*	2 (4)
Infratemporal fossa	3 (13)	0 (0)	.*07*	3 (6)
Retrosigmoid	1 (4)	5 (21)	.*09*	6 (13)
Lateral rhinotomy	1 (4)	6 (25)	.*05*	7 (15)
Endonasal^b^	12 (52)	12 (50)	*0*.*88*	24 (51)
Extent of Resection				
Gross Total	3 (13)	13 (54)	.*003*	16 (34)
Partial	20 (87)	11 (46)		31 (66)

Results are shown either by Number (proportion) or Mean (range). *ICA* Internal Carotid Artery.

^a^ Statistical significance comparing Surgery&Proton *vs* Surgery only treatments, using chi-squared test for proportions and Mann-Whitney test for mean.

^b^ Expanded endonasal approach.

When the resection was partial (31 cases– 66%), the most common residual zone was the retro-carotid area in its petrous or clival portion, the jugular foramen or the orbit (65%, 10% and 7%, respectively). Overall, 23 patients (49%) had adjuvant PT (mean 4.7 months after surgery, range 1.6–9.8). One patient initially failed to follow-up before adjuvant PT explaining the 9.8 months delay. Out of the 23 patients, 22 (97%) had a petroclival location and 20 (87%) a partial surgical resection. Mean total dose (Gray Relative Biologic Effectiveness) was 70 Gy (range 66–71), mean GTV (Gross Tumour Volume) and CTV (Clinical Target Volume) were 25 (range 3–147) and 42 (7–226) respectively.

**Fig 1:**
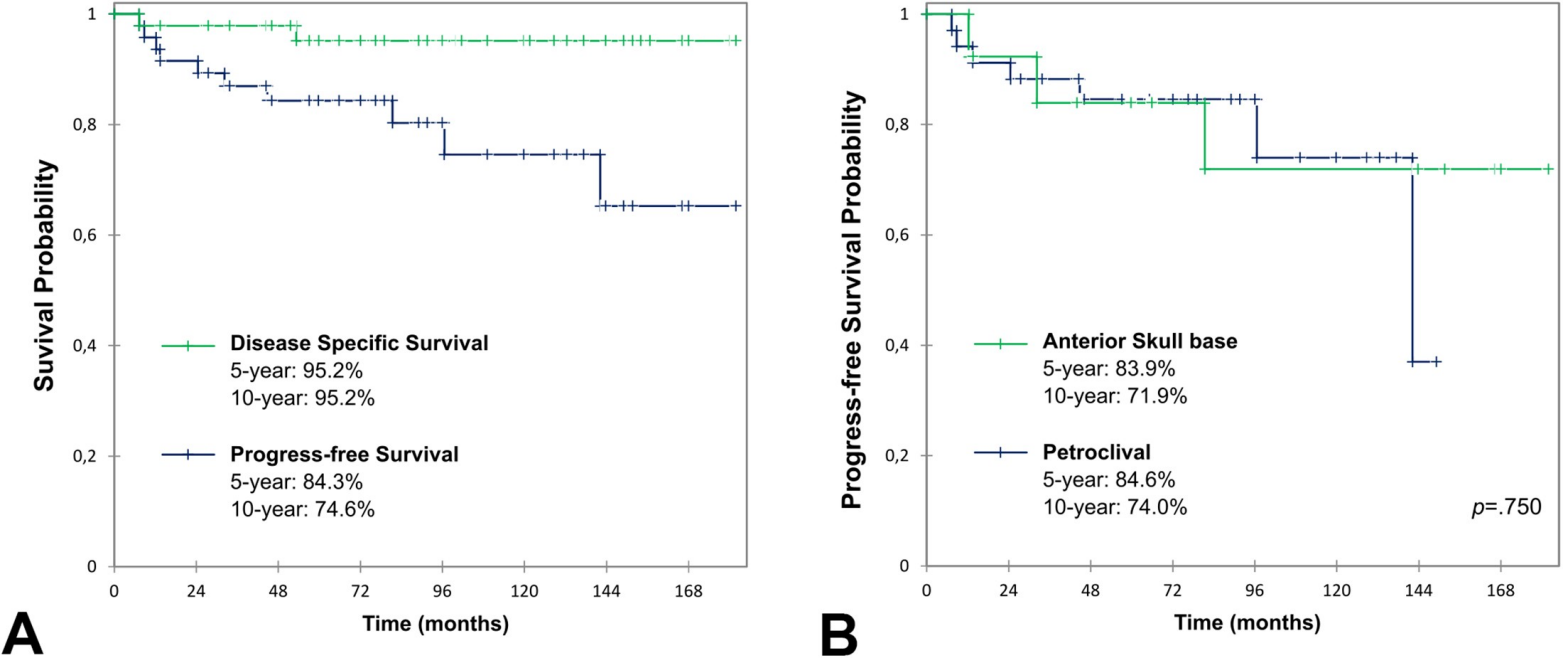
Overall and progress-free survival curve. Kaplan-Meier survival curves compared using log-rank test. A: Disease specific survival and Progress-free survival in the whole study population. B: PFS comparison of petroclival and anterior skull base anatomical locations shows no significant difference.

Overall, the mean clinical follow-up duration after the first surgical procedure was 91 months (7–182). Two patients were lost to follow-up at 14 and 52 months. Two patients died of non-tumoral (vascular) causes at 66 and 180 months. There were 2 disease-related deaths in the population, one due to post-operative complications at 7 months (cerebral abscess) and 1 due to CSA relapse at 53 months. Concerning the latter, the patient had a petroclival tumour which relapsed twice in a 3-month interval even though surgical GTR was achieved both times and before PT could be started. The 5- and 10-year DSS rates were both 95.2% (95% CI 88.5–100) (Fig 1A).

There were 9 cases of local relapse in 47 patients (19%), of which 8 who did not have initial adjuvant PT following surgery (Table 2). Thus, 8 (33%) of the initial 24 patients without initial adjuvant therapy presented with a local relapse.

**Table 2:** Characteristics of patients presenting with relapse.

Characteristics	Relapse	No relapse	*p*^a^
Number of patients	9 (19)	38 (81)	-
Age (years)	52 (21–77)	46 (10–85)	.*37*
Anatomic location			
Petroclival	6 (67)	27 (71)	.*80*
Anterior skull base	3 (33)	10 (29)	
Initial treatment			
Gross Total	4 (44)	11 (29)	.*37*
Partial Resection	5 (56)	27 (71)	
Proton therapy	1 (11)	22 (59)	.*01*
Follow-up			
Time to relapse (months)	51 (9–142)	-	
Secondary surgery	8 (90)	-	
Secondary proton therapy	5 (60)	-	
Disease-related death	1 (20)	-	
Total follow-up (months)	109 (52–180)	87 (7–182)	.*22*

Results are shown either by Number (proportion), or Mean (range).

^a^ Statistical significance comparing patients with and without relapse, using chi-squared test for proportions and Mann-Whitney test for mean.

**Fig 2:**
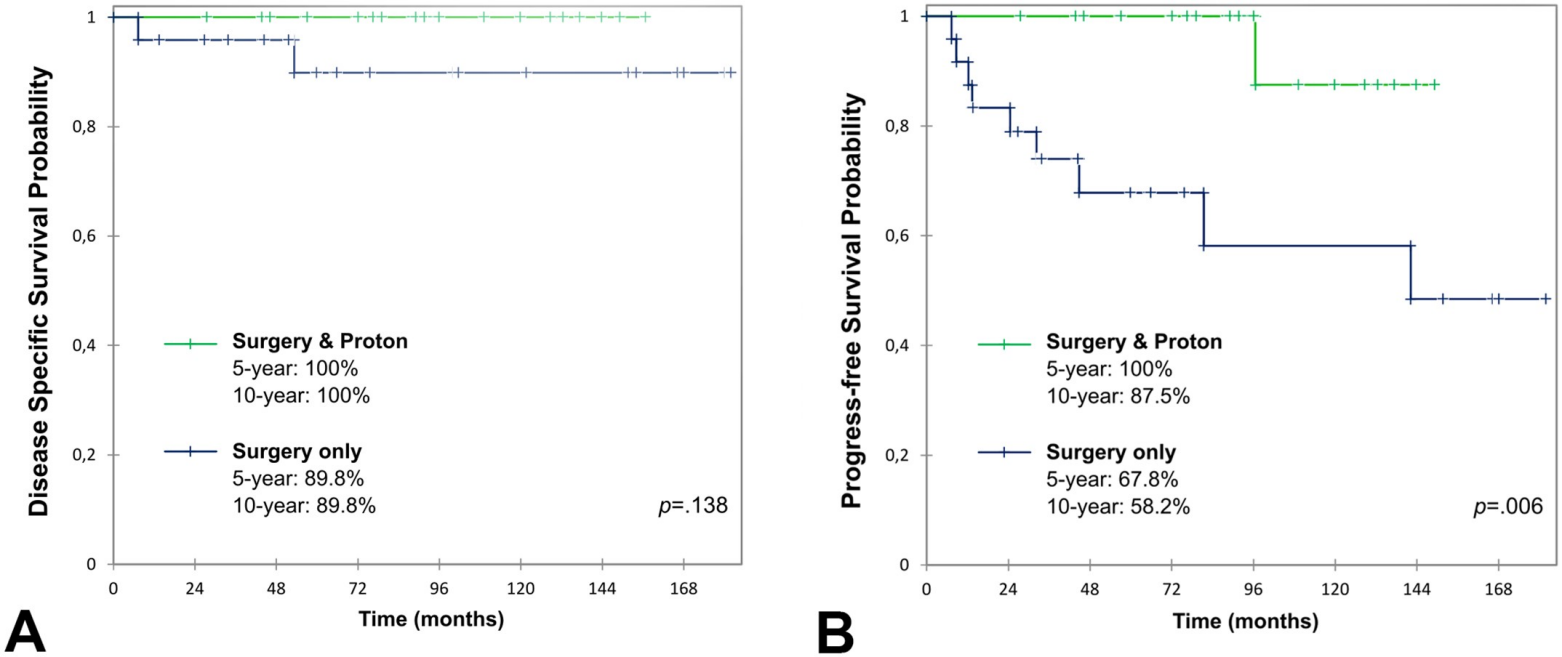
Survival curves comparing surgery only vs surgery and protontherapy. Kaplan-Meier survival curves compared using log-rank test: comparison of surgery and adjuvant proton therapy versus surgery only in the initial treatment. A: Disease specific survival showed no significant difference. B: Progress-free survival showed a significantly better outlook when surgery was associated with adjuvant proton therapy.

There were no regional or distant metastases. The 5- and 10-year PFS rate were 84.3% (95% CI 73.6–95.1) and 74.6% (95% CI 58.5–90.6), respectively (Fig 1A). When comparing surgery only to surgery and adjuvant PT, 5-year and 10-year DSS rate did not show any significant difference, 89.8% (95% CI 76.2–100) and 100%, respectively (*p* = 0.14, Fig 2A). There was however a significant difference in PFS: 5-year PFS were 67.8% (95% CI 47.7–88.0) and 100%; 10-year PFS were 58.2% (95% CI 33.5–82.8) and 87.5% (95% CI 64.6–100), respectively (*p* = 0.006, Fig 2B). At the time of the last follow-up, no patient had any tumour progress and 24 patients (51%) had a clinical and MRI surveillance of residual tumour without any progress. The regression of the initial pre-treatment symptoms was total, partial or had not regressed in 23 (49%), 16 (34%) and 8 (17%) cases, respectively.

When comparing petroclival and anterior skull base locations, 5-year PFS were 84.6% (95% CI 72.0–97.1) and 83.9% (95% CI 63.4–100); 10-year PFS were 74.0% (95% CI 51.7–96.3) and 71.9% (95% CI 44.0–99.9), respectively (*p* = 0.750, Fig 1B).

DSS and PFS were also calculated only on the petroclival population (34 patients), which is particularly concerned by PT strategy. When comparing surgery only to surgery and adjuvant PT, DSS and PFS were both significantly decreased in the surgery only group. Concerning DSS, 5 and 10-year survival rates were 76.4% (95% CI 46.1–100) and 100% respectively (*p* = 0.028). Concerning PFS, 5-year survival rates were 50.0% (95% CI 15.4–84.6) and 100%; 10-year rates were 50.0% (95% CI 15.4–84.6) and 85.7% (95% CI 59.8–100), respectively (*p* = 0.001).

Surgical and PT complications were reviewed and classified according to their severity using the CTCAE v4 in Table 3. PT induced significantly more complications than surgery (68% *vs* 26%, *p* < 0.001), but differences in severe complications (CTCAE ≥ 3) were not significant (25% *vs* 11%, *p* = 0.10). The main complications were cranial nerve palsy (19%) and cerebro-spinal fluid (CSF) leak (13%) due to surgery and hearing loss due to PT (39%). Other long-term complications specifically related to PT included vision loss, hypopituarism and temporal lobe radionecrosis in 11%, 18% and 18% of cases, respectively.

**Table 3:** Surgery side-effects compared to proton therapy side effects.

Characteristics	Surgery	Proton therapy	*p*^a^	All patients
Number of patients	47	28^b^	-	47
Presence of any complications	12 (26)	19 (68)	*<*.*001*	25 (53)
Death	1 (2)	0 (0)	.*44*	1 (2)
CTCAE ≥ 3	5 (11)	7 (25)	.*10*	10 (21)
CSF leak	6 (13)	-	-	6 (13)
Meningitis	4 (9)	-	-	4 (9)
Cranial nerve palsy	9 (19)	3 (11)	.*34*	11 (23)
Sensorineural HL	3 (6)	11 (39)	*<*.*001*	14 (30)
Conductive HL	2 (4)	3 (11)	.*28*	5 (11)
Severe HL^c^	2 (4)	6 (21)	.*02*	8 (17)
Dizziness	0 (0)	4 (14)	.*008*	4 (9)
PE	1 (2)	-	-	1 (2)
Vision loss	-	3 (11)	-	3 (6)
Hypopituarism	-	5 (18)	-	5 (11)
Temporal lobe radionecrosis	-	5 (18)	-	5 (11)

Results are shown either by Number (proportion), or Mean (range). There were no vessel injuries during surgery. *CTCAE* Common Terminology Criteria for Adverse Effects v4, *CSF* Cerebro-Spinal Fluid, *HL* Hearing loss, *PE* Pulmonary Embolism.

^a^ Statistical significance comparing surgery and proton therapy induced complications, using chi-squared test for proportions and Mann-Whitney test for mean.

^b^ Total number of patients having received Proton therapy (23 in the primary treatment and 5 in the secondary)

^c^ Hearing loss requiring treatment, or cophosis (≥grade 3 CTCAE v4), including conductive and sensorineural causes.

Using logistic regression, no significant factor for local relapse was found. The main significant toxicity factors were open dura surgery and PT (Table 4).

**Table 4:** Correlations of complication and local relapse risk, logistic regression.

	Toxicity risk		Relapse risk	
	*Coefficient*^a^	*p*	*Coefficient*^a^	*p*
Regression Model significance^b^		*<*.*001*		.*30*
Age	- 0.01 [-0.01; 0.01]	.*59*	0.40 [-0.32; 1.12]	.*27*
Sex^c^	- 2.16 [-4.87; 0.54]	.*12*	0.20 [-0.37; 0.77]	.*49*
Tumour size	- 0.01 [-0.01; 0.01]	.*02*	0.17 [-0.32; 0.80]	.*61*
Petroclival location^d^	7.05 [-0.28; 14.38]	.*06*	- 0.13 [-0.81; 0.55]	.*71*
Distant from ICA	0.54 [-2.92; 4.00]	.*76*	0.65 [-0.12; 1.43]	.*10*
Open Surgery	2.21 [-0.76; 5.18]	.*14*	- 0.03 [-0.60; 0.55]	.*93*
Dura opened	8.54 [5.23; 11.86]	*<*.*001*	0.42 [-0.16; 1.00]	.*16*
Partial Resection	1.30 [-2.70; 5.30]	.*52*	- 0.09 [-0.67; 0.48]	.*75*
Proton therapy	8.07 [3.82; 12.32]	*<*.*001*	0.58 [-0.20; 1.37]	.*14*

Relapse includes relapse and death; the correlation was calculated using a binary logistic regression test. Complications induced by surgery or radiotherapy were graded from 0 to 5 using the CTCAE v4 and the highest was noted for each patient, the correlation was calculated using an ordinal logistic regression test. Relapse risk was calculated using a binary logistic regression test. *ICA* Internal carotid artery, *OAT* Organs at risk near the Clinical Target Volume in proton therapy.

^a^ Standardized coefficient and 95% confidence interval.

^b^ Likelihood Ratio Test.

^c^ the fact to be a female (compared to male).

^d^ compared to anterior skull base.

## Discussion

This study on 47 patients tends to show that although very satisfying local control can be achieved when using the full therapeutic arsenal (surgery and adjuvant PT), the cost for the patient can be high with frequent side effects. These retrospective results do not seem to show any increased death rate with or without adjuvant proton therapy in the general skull base CSA population.

The main challenge in CSA management is to correctly balance toxicity risk versus local relapse risk. Although grade III and mesenchymal CSA subtypes drastically increase the mortality rate with aggressive local relapse and metastasis, the majority of the tumours are grade II with excellent long term survival and recent studies show 10-year survival ranging from 70% to 93% [3, 5–8, 10, 11, 17], which concur with our results.

There are many retrospective studies on petroclival CSA [3, 7, 8, 10, 11, 18–23]. Concerning CSA of the anterior skull base, there are however to our knowledge only case reports and a systematic review [9, 24–30]. There were some differences due to the different anatomical locations. As in the literature, the main symptom of petroclival CSA was the VI^th^ cranial nerve palsy causing diplopia in 48% of cases [20] (50% in our study). In our series, PT was used significantly less in the anterior CSA mainly because gross resection was more frequent and endoscopic clinical follow-up was easier in that location. Also, air cavity in the nasal fossa influences negatively proton dosimetry whereas, in the case of a relapse, surgical excision is straightforward. With this in mind, DSS and PFS was calculated for petroclival locations only showing a significant difference in both cases. Although the population was reduced inducing a very wide confidence interval, the significant difference in DSS without PT means the surgery only strategy in petroclival locations should be discussed and chosen cautiously.

Concerning disease-related deaths, our study did not find a significant difference when adjuvant PT was used or not. In the literature, some authors argue that PT can decrease the mortality rate, however they include patients treated up to 30 or more years ago (less efficient surgical treatment, possible lack of immunopathological confirmation of the diagnosis) and also include mesenchymal and grade III tumours (both increasing the death ratio) [3, 6–9]. We deliberately chose to limit our inclusion criteria to a timeframe when endoscopic surgery and PT were routinely available and to grade I or II tumours, so as to have more comparable groups. More recent papers find results similar to ours, with PT affecting only the PFS [10, 11].

To prevent local relapse in skull base CSA, most authors agree that surgery combined to adjuvant radiotherapy is superior to surgery only [3, 7, 8, 10, 11, 20, 29, 31–33]. PT seems to be the best strategy, as the physical properties of protons enable precise irradiation of large areas, limiting damage of adjacent neural structures [12, 34, 35]. Although photon radiosurgery seems to have good results on smaller lesions [36–39], most authors have shown that protons are superior to treat large CSA tumours [13, 40, 41].

Our study showed that 33% of patients without adjuvant PT presented a local relapse, compared to 40% and 44% in recent studies of petroclival CSA [10, 42]. We showed that the absence of PT was the only significant factor found to increase local relapse. Also, we found significant decrease of PFS when the patient did not undergo adjuvant PT although this population was more likely to have had GTR. Indeed, our study did not show any significant PFS difference between patients with GTR or partial resection. This tends to confirm that GTR is not a necessary objective if surgeons feel an increased surgical risk is being taken. Many authors who failed to obtain GTR also showed good long term results, especially when associated to adjuvant therapy [6, 11, 22, 31].

The two main significant factors leading to treatment complications were surgery involving dura opening and PT. Due to the tumour location, dura opening is sometimes necessary, but as said previously, excessive total resection should be avoided to limit the risk of complications and especially CSF leak. Moreover, these procedures should take place in highly trained skull base units. Toxicity rate was also significantly higher in the population having received PT. PT toxicity has been well studied in the literature. The most severe complications are temporal lobe necrosis [43] and optic neuropathy [12, 17, 34]. The most frequent complication concerned the inner ear (sensorineural hearing loss) due to the fact that there are no dose constraints on the ipsilateral cochlea (constraint of 55 GyRBE only on the contralateral ear) to optimise coverage of target volumes [12]. In our study, 25% of patients who underwent PT presented ≥3 CTCEA complications, compared to 7 and 14% in two studies, which had larger populations (159 and 77) and focused on PT [12, 17]. These complications are considered acceptable by most authors due to the malignant nature of CSA. However concerning low-grade tumours, we believe surgery only should be discussed with the patient due to the absence of metastases and long delay before local relapse (51 months in our study, 42 months in a recent study [10]).

Regarding the long relapse delay of CSA and frequent toxicity of PT, it could be argued that PT should not systematically be used as an adjuvant treatment, moreover it does not significantly decrease the death rate. However, a study seems to show that PT could give better results on primary compared to recurrent tumours [12]. Also, given the high-grade toxicity due to surgery (grade 5, one death in our series), a PT only strategy could be discussed [42]. In this literature review, studying 560 cranial CSA patients (grade I, II and III treated from 1980 to 2010), 5-year disease-free rate was 56%, 91% and 81% for surgery only, surgery and radiotherapy and radiation only, respectively (67.8% and 87.5% for surgery only and surgery and PT in our series). Further studies should be conducted on the PT only strategy which seems to offer a high 5-year disease-free rate and lower toxicity than when combined to surgery.

The main limit of this study is the retrospective design due to the rarity of skull base CSA. Also, the small number of patients implies that the results need to be compared to the literature for validity, especially concerning the PT strategy, as prospective controlled studies will be very difficult to set up requiring many patients and long follow-up.

Our retrospective study cannot give a clear-cut treatment algorithm and CSA management should be decided on a case-by-case basis. We would tend to recommend a strategy reducing toxicity for grade I and II patients. Indeed, with current surgical and PT standards, cranial CSA seem to have an excellent long-term DSS rate and local relapses can be controlled. Keeping this in mind, we believe that the surgery only strategy can be discussed with patients who do not wish to risk PT induced toxicity. Concerning surgery, procedures should clearly only take place in highly trained skull base units and remain conservative. Strategies for which we do not have any results in our study, such as PT only, should also be studied to reduce toxicity. In any case, patients should comply to a close clinical and MRI follow-up for a prolonged period (five to ten years). More severe and life-threatening grade III and mesenchymal CSA should however receive the full-scale surgery and PT treatment.

## Conclusion

The results of this retrospective study support the rationale of a conservative approach in low-grade CSA to limit toxicity. During surgery, partial resection should be considered in case of close proximity with major anatomical structures, taking great care to limit the risk of CSF leakage and vascular damage. PT should be discussed with the patient especially in cases of GTR when a surgery only strategy could be considered. Cases with petroclival CSA must be discussed cautiously, as they are most prone to PT side-effects (especially cochlea-vestibular toxicity) but could also benefit most from PT against relapse and for a significantly higher long-term survival rate. Discussion on a case-by-case basis within a multidisciplinary team to best assess the toxicity and relapse risk ratio is paramount.

## Supporting information

S1 Dataset Complete dataset from the patients described in this article. (XLSX)
